# A modular positive feedback-based gene amplifier

**DOI:** 10.1186/1754-1611-4-4

**Published:** 2010-02-26

**Authors:** Goutam J Nistala, Kang Wu, Christopher V Rao, Kaustubh D Bhalerao

**Affiliations:** 1 Department of Agricultural and Biological Engineering, University of Illinois at Urbana-Champaign, 1304 W Pennsylvania Ave, Urbana, IL, 61801, USA; 2 Department of Chemical and Biomolecular Engineering, University of Illinois at Urbana-Champaign, 600 S Mathews Ave, Urbana, IL, 61801, USA

## Abstract

**Background:**

Positive feedback is a common mechanism used in the regulation of many gene circuits as it can amplify the response to inducers and also generate binary outputs and hysteresis. In the context of electrical circuit design, positive feedback is often considered in the design of amplifiers. Similar approaches, therefore, may be used for the design of amplifiers in synthetic gene circuits with applications, for example, in cell-based sensors.

**Results:**

We developed a modular positive feedback circuit that can function as a genetic signal amplifier, heightening the sensitivity to inducer signals as well as increasing maximum expression levels without the need for an external cofactor. The design utilizes a constitutively active, autoinducer-independent variant of the quorum-sensing regulator LuxR. We experimentally tested the ability of the positive feedback module to separately amplify the output of a one-component tetracycline sensor and a two-component aspartate sensor. In each case, the positive feedback module amplified the response to the respective inducers, both with regards to the dynamic range and sensitivity.

**Conclusions:**

The advantage of our design is that the actual feedback mechanism depends only on a single gene and does not require any other modulation. Furthermore, this circuit can amplify any transcriptional signal, not just one encoded within the circuit or tuned by an external inducer. As our design is modular, it can potentially be used as a component in the design of more complex synthetic gene circuits.

## Background

Positive feedback is a common mechanism involved in the regulation of genetic circuits [1]. Any time a gene product has the capacity to enhance its own production, either directly or indirectly, the circuit is said to involve positive feedback. A number of behaviors can be attributed to positive feedback loops. The defining one is clearly amplification. More complex behaviors include bistability and hysteresis. In addition, positive feedback is an integral element in many oscillatory, pattern-formation, and intracellular polarization processes [2,3].

In a number of synthetic biology applications, positive feedback has been used to design switches, oscillators, and amplifiers. Besckei and coworkers [4], for example, showed in yeast that a simple positive feedback loop could transform a graded response to an inducer into a binary one. Likewise, Kramer and Fussenegger [5] showed that positive feedback could be used to generate hysteresis with respect to an inducer in mammalian cells. Maeda and Sano [6] analyzed a synthetic positive feedback loop in *E. coli *and demonstrated that it could give rise to either a graded or hysteretic response depending on the specific configuration. In terms of building circuits, Ajo-Franklin and coworkers [7] demonstrated that positive feedback could be used to engineer memory into yeast cells. Stricker and coworkers [8], on the other hand, built a simple oscillator by coupling positive feedback with negative feedback. In work most closely related to the present study, Sayut and coworkers [9,10] demonstrated that a positive feedback loop could make the transcriptional activity of the quorum-sensing regulator LuxR more sensitive to autoinducer. In these regards, their design is most closely related to how positive feedback is typically employed in electronic circuits, namely to amplify the response to a signal.

In this work, we constructed a modular genetic amplifier in *Escherichia coli *based on a constitutively active, autoinducer-independent variant of the quorum-sensing regulator LuxR from *Vibrio fischeri *[11]. Our goal was to develop a simple network component that could be coupled to any cell-based sensing system where the output involves the transcription of some gene. In these regards, we sought to engineer an "off the shelf" device that could be readily implemented in any gene circuit. To test the ability of this device to amplify a transcriptional output, we coupled our device to a one-component tetracycline sensor and a two-component aspartate sensor. In both cases, we found that our amplifier was able to increase the sensitivity to the input signal and intensify the output signal.

## Methods

### Media, growth conditions, and bacterial strains

All cultures experiments were performed in either Luria-Bertani (LB) broth (tryptone: 10 g/L, yeast extract: 5 g/L, and NaCl: 10 g/L) or M9 minimal media supplement with 0.4% glucose, 1 μg/mL thiamine, and 1 μg/mL biotin. All experiments were performed at 37°C unless noted otherwise. Antibiotics were used at the following concentrations: ampicillin at 100 μg/mL, chloramphenicol at 20 μg/mL, and kanamycin at 40 μg/mL. Primers were purchased from IDT Inc. (Coralville, IA). Restriction enzymes were purchased from New England Biolabs Inc. (Ipswitch, MA) and Fermentas Inc. (Glen Burnie, MD) and used according to the manufacturer's recommendations.

All cloning steps were performed in *E. coli *strain DH5α. Subsequent experiments involving anhydrotetracycline induction were conducted in *E. coli *strain GN100 (F^- ^*ilvG rfb-50 rph-1 *Δ*envZ*::FRT attB_λ_::[P_N25_-*tetR lacI*^q ^*spcR*]) and those involving aspartate induction were performed in GN101 (F^- ^*ilvG rfb-50 rph-1 *Δ*envZ*::FRT). Strain GN100 was constructed first by P1*vir *transduction of the Δ*envZ*::*kan *insert from JW3367-3 (The *E. coli *Genetic Stock Center, CGSC# 10509) into MG1655. The antibiotic cassette from the FRT-Kan-FRT insert was then removed by transformation of pCP20 into the strain and selection on ampicillin at 30°C [12]. Loss of the helper plasmid pCP20 was obtained by growth at 42°C under non-selective conditions on LB agar. Lastly, the chromosomally integrated TetR/LacI expression cassette from DH5αZ1 [13] was moved into this strain by P1*vir *transduction, yielding GN100. Similarly, strain GN101 was constructed in an identical manner except that it does not harbor the TetR/LacI expression cassette from DH5αZ1.

### Plasmids Construction

Table 1 provides a list of the plasmids used in this study. The plasmid pPROTetE-Kan-p15A was made by swapping the ColE1 origin of pPROTet.E with the p15A origin from pZA34-luc using the restriction sites XbaI and SacI and by swapping the chloramphenicol resistance gene with the kanamycin resistance gene from pZE21 using the restriction sites XhoI and SacI. The plasmid pPROTetE-Amp was made by replacing the chloramphenicol resistance gene in pPROTet.E with the ampicillin resistance gene from pZE12 using the restriction sites XhoI and SacI.

**Table 1:** Plasmids used in this study

Plasmid	Relevant characteristic	Reference
pTJ003	*bla *P_*lpp*_-*taz *ori p15A	[33]

pPROTet.E	*cm *P_*LtetO-1 *_ori ColE1	Clontech

pPROBE-GFP	*kan GFP[tagless] *ori p15A	[15]

pZE12-luc	*bla *P_*LlacO-1*_-*luc *ori ColE1	[13]

pZE21	*kan *P_*LtetO-1 *_ori ColE1	[13]

pZS24	*kan *P_*lac/ara-1 *_ori pSC101	[13]

pluxRI	*cm *P_*lac/ara-1*_-*luxR-luxI *ori ColE1	[14]

pPROTetE-kan-p15A	*kan *P_*LtetO-1 *_ori p15A	

pGN3	*kan *P_*LtetO-1*_-luxR * ori p15A	

pGN11	*kan *P_*LtetO-1*_-*luxRΔ*_*2-156 *_ori p15A	

pGN12	*kan *P_*LtetO-1*_-*luxRΔ*_*2-162 *_ori p15A	

pGN23	*cm *P_*lux *_ori ColE1	

pGN62-Kan	*kan *P_*OmpC*_-*luxRΔ_2-162 _*ori p15A	

pGN68	*cm *P_*lux *_-*GFP [tagless]*-*luxRΔ_2-162 _*ori ColE1	

pGN69	*cm *P_*lux*_-*GFP[tagless] *ori ColE1	

pPROTetE-amp	*bla *P_*LtetO-1 *_ori ColE1	

pGN76	*bla *P_*LtetO-1*_-*taz *ori ColE1	

pGN77	*bla *P_*LtetO-1*_-*taz *ori pSC101	

Plasmids are from this study unless noted otherwise.

The *luxI-GFP *transcriptional fusion was made first by PCR amplification of the *luxI *promoter using the plasmid p*lux*GFPuv [14] as the template with the primers KW134F (CAG ATA TCG ACG TCA GTC C) and KW134R2 (ATA GAA TTC TGC GTT TAT TCG ACT ATA AC). The resulting fragment was then cloned into the plasmid pPROTet.E using the restriction sites EcoRI and AatII, yielding the plasmid pGN23. The green fluorescent protein (GFP) was PCR amplified from pPROBE-*gfp*[tagless] [15] using primers GN10F (GGG GAA TTC ATA CGT ATT TAA ATC AGG AGT GGA AAT GAG TAA AGG AGA AGA ACT T) and GN10R (GGG GGA TCC TTA TTA TTT GTA TAG TTC ATC CA). The resulting fragment was then cloned into the EcoRI and BamHI restriction sites of the pGN23, yielding the plasmid pGN69.

The LuxR* (LuxR[A221V]) expression plasmids were constructed using two rounds of PCR. In the first round, the *luxR *gene was amplified with primers KW78F1 (AAC TTT ATA AGG AGG AAA AAC ATA TGA AAA ACA TAA ATG CCG AC) and KW078R (ACT GTC GAC TTA ATT TTT AAA GTA TGG GC) using pLuxRI [14] as the template. The resulting product was then used as a template for a second round of PCR this time using primers KW078F2 (TAT GAA TTC AAC TAA AGA TTA ACT TTA TAA GGA GGA AAA ACA) and KW078R. It was then digested with EcoRI and SalI and sub-cloned into the EcoRI and SalI cut-sites of pPROTetE-Kan-p15A. Enzymatic inverse PCR was used to introduce the Ala221Val (GCG- > GTG) point mutation in the *luxR *gene with primers KW079F (ATA GGT CTC TGT GCA AAT GAA ACT CAA TAC AAC) and KW079R (ATA GGT CTC TGC ACA TTG GTT AAA TGG AAA GTG A). The resulting PCR product was then digested with BsaI and ligated to obtain pGN3.

The *luxR*Δ_2-156 _expression plasmid was also constructed using two rounds of PCR. The *luxR *gene was first amplified with primers KW112F (AAC TTT ATA AGG AGG AAA AAC ATA TGA ACA TAC CAT TAA TTG TTC C) and KW078R using pLuxRI as the template. The resulting PCR product was then amplified using primers KW078F2 and KW078R. It was then cloned into the EcoRI and SalI cut-sites of pPROTetE-Kan-p15A, yielding pGN11. Likewise, the *luxR*Δ_2-162 _expression plasmid was made by amplifying the *luxR *gene with primers KW113F (CTT TAT AAG GAG GAA AAA CAT ATG CCT TCT CTA GTT GAT AAT TAT C) and KW078R using pLuxRI as the template. The resulting product was amplified again as before using primers KW078F2 and KW078R. The PCR product was then digested with EcoRI and SalI and sub-cloned into the EcoRI and SalI cut-sites of pPROTetE-Kan-p15A, yielding pGN12.

The positive-feedback module was constructed using two rounds of PCR. In the first round, the primers GN09F2 (AAC TAA AGA TTA ACT TTA TAA GGA GGA AAA ACA TAT GCC TTC TCT AGT TGA TAA T) and KW171R (AAT AGC GGC CGC TTA TTA ATT TTT AAA GTA TGG GC) were used to amplify the luxRΔ_2-162 _domain [16] using pLuxRI [14] as the template. The resulting PCR product was then used as template for a second round of PCR this time using primers GN09F (GGG GGA TCC AAC TAA AGA TTA ACT TTA TAA GGA GGA AAA ACA T) and KW171R (AAT AGC GGC CGC TTA TTA ATT TTT AAA GTA TGG GC). The resulting fragment was then digested with BamHI and NotI and sub-cloned into pGN69, yielding pGN68.

The aspartate positive feedback module was constructed first by amplifying the P_*ompC *_promoter (genomic region 2310762-2310962) using primers GN03F (GGG CTC GAG GTT CCC TTG CAT TTA CAT TTT) and GN05R (GGG GAA TTC TAA CTT TCA TGT TAT TAA CCC). The PCR product was then digested with XhoI and EcoRI and sub-cloned into the respective sites of pPROTetE-Kan-p15A, thus replacing the native P_LtetO-1 _promoter with the P_*ompC *_promoter. The primers GN06F2 (GGG GTC GAC ATG CCT TCT CTA GTT GAT AA) and KW171R were used to amplify luxRΔ_2-162 _using pGN68 as the template. The resulting PCR product was digested with SalI and NotI and then sub-cloned into the respective sites of pPROTetE-Kan-p15A, yielding pGN62.

The aspartate sensor module was constructed first amplifying the *taz *gene from pTJ003 using the primers GN13F (GGG GAA TTC TTA AAG AGG AGA AAG GTA CCC ATG ATT AAC CGT ATC C) and GN12R (GGG GTC GAC TTA CCC TTC TTT TGT CGT GCC CT). The PCR product was then digested with EcoRI and SacI and cloned into the unique respective restriction sites, yielding pGN76. The ColE1 origin in pGN76 was then replaced with the pSC101 origin from the pZS24 plasmid using the restriction sites AvrII and SacI, yielding pGN77.

### Fluorescence Assays

To measure fluorescent protein expression, cultures were first grown overnight and then subcultured to an OD_600 _of 0.05 in fresh media. The cultures were first allowed to grow to an OD_600 _of 0.20, at which point the inducer was added. The cultures were then grown overnight prior to taking the measurements. 100 μL of the culture was then transferred into a 96 well microplate, and the relative fluorescence and optical density at 600 nm (OD_600_) were measured using a Tecan Safire2 microplate reader. The fluorescence readings, given as relative fluorescence units (RFU), were normalized with the OD_600 _absorbance to account for cell density. All experiments were performed in triplicate with 95% confidence intervals reported.

## Results and Discussion

### Design of positive-feedback amplifier

In order to construct a positive feedback circuit, we required a transcriptional activator that did not interfere with native gene regulation in *E. coli*. In addition, we required that the activator be constitutively active and not dependent on the addition of an exogenous inducer. Given these constraints, we chose the LuxR protein from *Vibrio fischeri *[11]. This protein, normally involved in quorum sensing and bioluminescence, activates the transcription of the *luxIADCBE *operon in response to acyl homoserine lactone (AHL). AHL binding stabilizes the LuxR dimer and, as a result, increases its ability to activate transcription [17-19].

While wild-type LuxR does not appear to interfere with native *E. coli *regulation, it still requires an exogenous inducer. However, a number of approaches exist for making constitutively active derivatives of LuxR and thus satisfying our design constraints. For example, an Ala221Val point mutation was previously found to constitutively activate LuxR [20]. The alanine at position 221 enables the N-terminal signaling domain to inhibit the activity of the C-terminal, DNA-binding domain. Presumably, mutating this residue to a valine prevents the N-terminal domain from interfering with DNA binding. Consistent with this model, deleting the N-terminal domain of LuxR was also found to yield a constitutively active variant [21,22].

**Figure 1:**
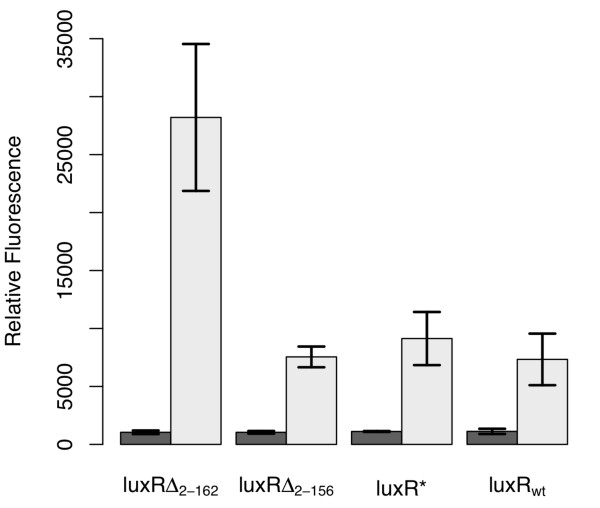
**Comparison of constitutive LuxR variants**. In these experiments, LuxR was expressed from a tetracycline-inducible promoter, P_*LtetO-1*_, in strain GN101, which harbors a chromosomal copy of *tetR*. Activity was determined by the ability of these different variants to induce expression from the P_*luxI *_promoter, using GFP as the readout, in the absence of any autoinducer. Dark bars denote the uninduced case and light bars the induced case (200 ng/mL aTc). Error bars denote 95% confidence intervals.

Based on these previous studies, we engineered three constitutively active variants of LuxR to test their suitability in designing an amplifier. The first, denoted by LuxR*, harbors the Ala221Val point mutation. The other two, denoted by LuxR_Δ2-156 _and LuxR_Δ2-162 _respectively, involved different N-terminal deletions, where the subscript denotes the deleted fragment. To test the relative effectiveness of these three different constitutive LuxR variants, we determined how strongly they could activate expression from the P_*luxI *_promoter, using the green fluorescent protein (GFP) as our transcriptional readout. The results from these experiments are shown in Figure 1. All of the LuxR variants, including the wild-type control, were able to induce expression from the P_*luxI *_promoter. Of the three, only LuxR_Δ2-162 _was capable in our hands of enhancing transcription relative to the wild-type control. Based on these results, we chose to use the LuxR_Δ2-162 _variant to design the amplifier.

**Figure 2:**
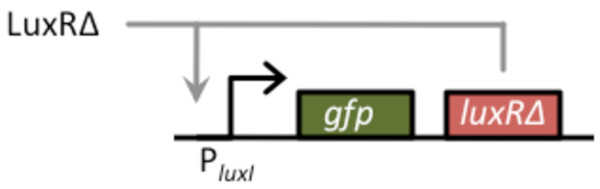
**Schematic of positive-feedback amplifier**. The basic design for the amplifier consists of GFP and LuxR_Δ2-162 _arranged in a bicistronic configuration under the control of the P_*luxI *_promoter. LuxR_Δ2-162 _functions in a positive feedback loop as it can bind to the P_*luxI *_promoter and activate its own transcription. In our design, LuxR_Δ2-162 _is also used as the input signal for the amplifier. LuxR_Δ2-162_, therefore, functions both as the input and positive feedback signal. GFP, the output signal, provides a measure of transcriptional activity.

To construct the amplifier, we cloned GFP and LuxR_Δ2-162 _in a bicistronic configuration behind the P_*luxI *_promoter on high-copy number plasmid (ColE1 origin of replication). In this arrangement, LuxR_Δ2-162 _functions in a positive feedback loop as it can bind to the P_*luxI *_promoter and activate its own transcription (Figure 2). The reason we cloned LuxR_Δ2-162 _downstream of the GFP reporter is to control for polar effects when we compared results involving positive feedback to those lacking it. To induce this circuit, we again used LuxR_Δ2-162_, this time as the input signal. In such a design, the output of the sensor is LuxR_Δ2-162_, which in turn feeds back into the amplifier. In these regards, LuxR_Δ2-162 _is used both as the input and positive feedback signal. For the output, we used GFP as it provides a facile measure of transcriptional activity. This choice is in no way limiting, and any gene can in practice be used as the output.

### Validation of amplifier using a tetracycline sensor

**Figure 3:**
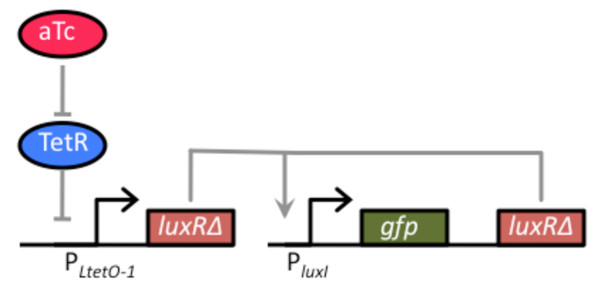
**Schematic of tetracycline sensor coupled to the positive-feedback amplifier**. The one-component tetracycline sensor consists of a plasmid where LuxR_Δ2-162 _has been cloned behind the TetR-regulated P_*LtetO-1 *_promoter. In the absence of the inducer anhydrotetracycline (aTc), dimeric TetR binds to the O2 operator sites within the P_*LtetO-1 *_promoter and represses transcription. However, when bound with aTc, TetR is no longer able to bind to the O2 operator sites within the promoter, thus enabling dose-dependent control of LuxR_Δ2-162_. This sensor was coupled with the positive feedback amplifier, encoded on a separate plasmid, by transforming cells (GN100) constitutively expressing a chromosomal copy of the *tetR *gene with the two plasmids respectively harboring the sensor and amplifier.

We first tested the amplifier by coupling it to a one-component tetracycline sensor (Figure 3). In this design, we cloned LuxR_Δ2-162 _behind the TetR-regulated P_*LtetO-1 *_promoter on a compatible, medium copy-number plasmid (p15A origin of replication) [13]. In the absence of the tetracycline analogue, anhydrotetracycline (aTc), dimeric TetR binds to the O2 operator sites within the P_*LtetO-1 *_promoter and represses transcription. However, when TetR is bound with aTc, it no longer binds and represses the P_LtetO-1 _promoter, enabling dose-dependent control of gene expression. Thus, the aTc-inducible promoter functions as a one-component tetracycline sensor with LuxR_Δ2-162 _as the output.

**Figure 4:**
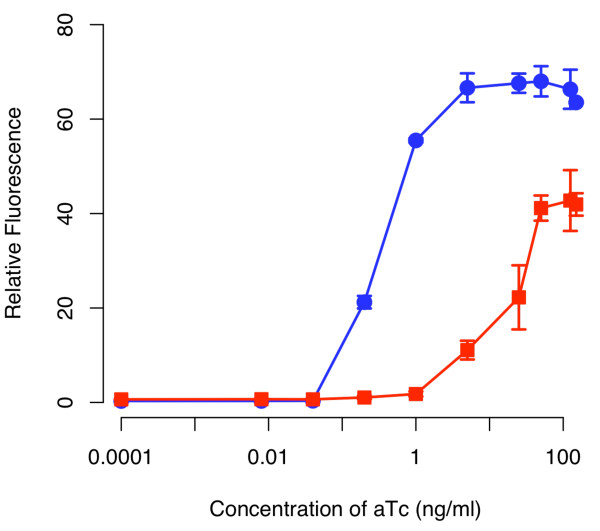
**Comparison of tetracycline sensor with positive feedback (solid circles) and without (solid square)**. Schematic of positive feedback design is shown in Figure 3. The design lacking positive feedback is otherwise identical to one with positive feedback except that only GFP is expressed from the P_*luxI *_promoter. In these experiments, cells were grown overnight at the indicated concentrations of aTc prior to measurements. The fluorescence values were normalized with the OD_600 _absorbance to account for cell density. Error bars denote 95% confidence intervals for the measurement average.

To couple this sensor with the amplifier, we transformed cells (GN100) constitutively expressing a chromosomal copy of the *tetR *gene with the two plasmids respectively harboring the sensor and amplifier (see Materials and Methods for details). A schematic of the integrated design is given in Figure 3. When we tested this design, we found that the amplifier increased both the sensitivity and dynamic range of the integrated circuit relative to an otherwise identical circuit lacking positive feedback (Figure 4). In particular, we found that positive feedback increased the sensitivity to aTc by roughly two orders of magnitude. In other words, we observed equivalent levels of expression in the circuit involving positive feedback at aTc concentrations roughly one hundred times less than those observed with the circuit lacking positive feedback. Moreover, we found that positive feedback increased the dynamic range by roughly 50%. By range, we mean the ratio of expression under saturating inducing (100 ng/ml aTc) and non-inducing (0 ng/ml aTc) conditions.

**Figure 5:**
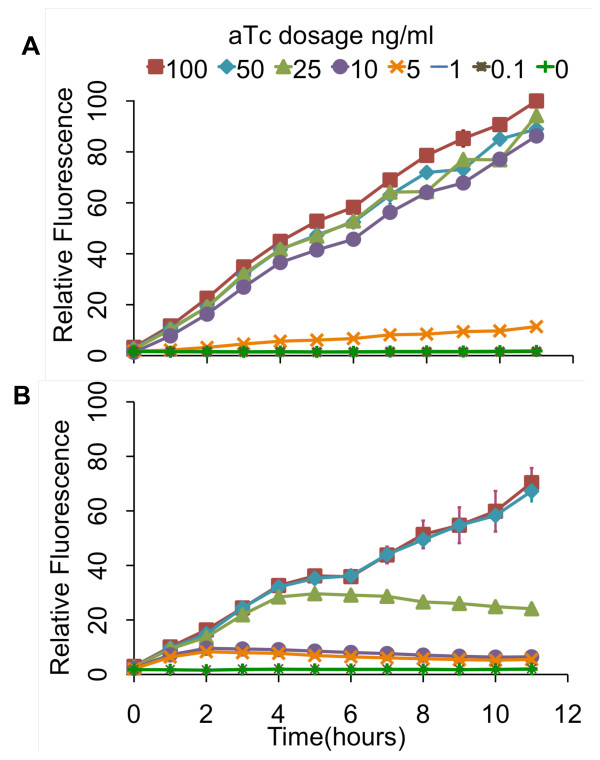
**Kinetic analysis of tetracycline sensor with positive feedback (A) and without (B)**. In these experiments, cells were grown for 12 hours at varying levels of aTc induction with measurements taken every hour. The fluorescence values were normalized with the OD_600 _absorbance to account for cell density. The scale for both sets of experiments is the same. Error bars denote 95% confidence intervals for the measurement average.

In addition to these endpoint measurements, we also performed kinetic experiments where we measured the response over a twelve-hour interval to varying concentrations of aTc (Figure 5). Consistent with our end-point measurements, we found that the design involving positive feedback was more sensitive to aTc and had a wider dynamic range of expression levels. Collectively, these results demonstrate that our genetic amplifier is capable of both increasing the sensitivity and dynamic range of this one-component tetracycline sensor.

We last tested whether the amplifier would endow the cell with memory. While not a design goal, multiple studies have shown that positive feedback can lead to bistability and hysteresis [1,5,23]. Therefore, we speculated that cells harboring the amplifier might be able to "remember" previous exposures to aTc. However, when we transferred cells from media containing aTc to media lacking it, we no longer observed any GFP expression relative to the background after we grew the cells up (data not shown). These results indicate the positive feedback loop involving LuxR_Δ2-162 _is able to amplify the response to an inducer but is incapable of sustaining the response in the absence of inducer.

Based on what we know about the properties of LuxR, specifically the role of AHL in stabilizing LuxR, the reason the circuit does not sustain activation is likely due to the protein dimer being degraded too quickly. In other words, we suspect that LuxR_Δ2-162 _dimer is being degraded at a rate greater than it is being produced by positive feedback alone (though we did not directly make this measurement). More specifically, positive feedback alone is unable to sustain the expression of LuxR_Δ2-162 _in the absence of some exogenous source, in our case the one-component sensor. That said, the positive feedback is still strong enough to amplify the response when an external input signal is present.

### Validation of amplifier using an aspartate sensor

**Figure 6:**
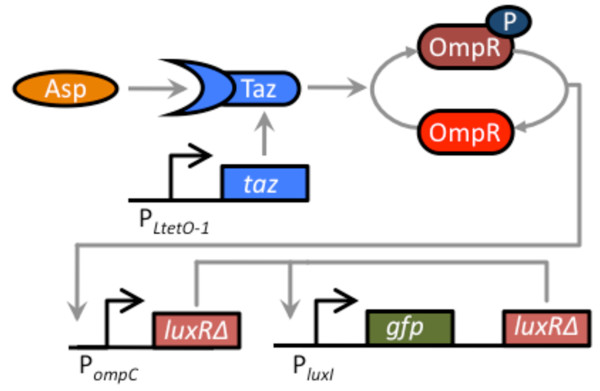
**Schematic of aspartate sensor coupled to the positive-feedback amplifier. **The two-component sensor consists of the Taz sensor kinase and the OmpR response regulator. Taz controls the level of phosphorylated OmpR (OmpR-P), which in turn activates the expression from the P_*ompC *_promoter. When the Taz sensor kinase is bound with aspartate, it increases the levels of OmpR-P, leading to increased expression from the P_*ompC *_promoter. In our design, the Taz sensor kinase has been cloned behind the constitutive P_*LtetO-1 *_promoter on one plasmid (the cells used in these experiments do not possess TetR). On a second plasmid, LuxR_Δ2-162 _has been cloned behind the P_*ompC *_promoter, resulting in the expression of LuxR_Δ2-162 _being aspartate dependent. The third plasmid harbors the positive feedback amplifier. The sensor was coupled to the amplifier by transforming the three plasmids into a Δ*envZ *null mutant (GN101).

We next tested the amplifier by coupling it to a two-component aspartate sensor (Figure 6). To do this, we used the hybrid Tar-EnvZ (Taz) sensor kinase [24]. This chimeric, transmembrane sensor kinase controls the levels of phosphorylated OmpR, which in turn activates the expression from the P_*ompC *_promoter. When the Taz sensor kinase is bound with aspartate, it increases the levels of OmpR-P, leading to increased expression from the P_*ompC *_promoter. In addition to amino acids, EnvZ chimeras have been constructed to sense other inputs such as sugars and light [25,26].

In order to couple the two-component aspartate sensor with our genetic amplifier, we cloned LuxR_Δ2-162 _behind P_*ompC *_promoter on a compatible, medium copy-number plasmid (p15A origin of replication). To introduce the Taz sensor kinase into *E. coli*, we cloned this gene behind the constitutive P_LtetO-1 _promoter on a compatible, low copy-number plasmid (pSC101 origin of replication). Note, these experiments were performed in cells lacking a chromosomal copy of the *tetR *gene, so the P_*LtetO-1 *_promoter in this background is constitutive. To construct the integrated circuit in *E. coli*, we transformed a Δ*envZ *null mutant (GN101) with these three plasmids.

**Figure 7:**
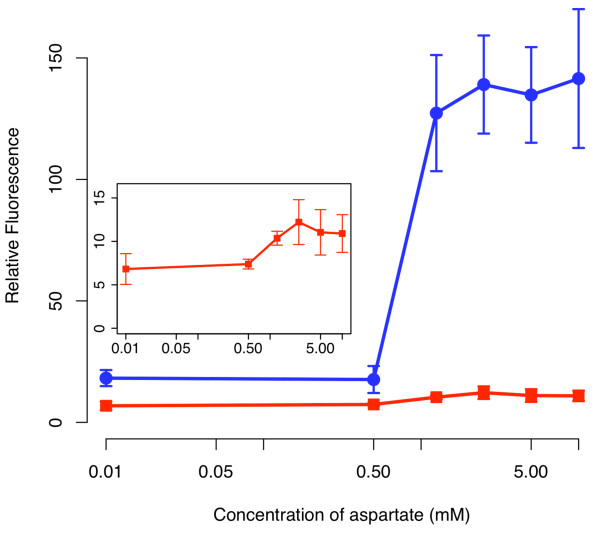
**Comparison of sensor output in the presence (solid circles) and absence (solid squares) of the positive feedback amplifier**. Schematic of positive feedback design is shown in **Figure 6**. The design lacking positive feedback is otherwise identical to one with positive feedback except that only GFP is expressed from the P_*luxI *_promoter. In these experiments, cells were grown overnight at the indicated concentrations of aspartate prior to measurements. The fluorescence values were normalized with the OD_600 _absorbance to account for cell density. Inset figure shows the magnification of the response for the design lacking positive feedback. Error bars denote 95% confidence intervals for measured averages.

Similar to what we observed with the one-component tetracycline receptor, we found that the amplifier increased both the range and sensitivity when coupled to the two-component aspartate sensor as compared to an otherwise identical circuit lacking positive feedback (Figure 7). Unlike the case with the one-component sensor, we observed only a minor increase in sensitivity. However, we observed a significant amplification of the response. In particular, the amplifier increased the dynamic range by roughly an order of magnitude whereas the sensitivity increased by approximately a factor of two. While these results demonstrate that the amplifier is modular as it can readily be applied to different sensor systems, they also demonstrate that the performance of the amplifier is context dependent. In particular, we observed mostly an increase in the range when the amplifier was coupled to the two-component aspartate sensor kinase and, conversely, mostly an increase in sensitivity when it was coupled to the one-component tetracycline sensor.

We note that we observed only weak activation, roughly two-fold, in response to aspartate in the absence of positive feedback. This level of activation is less than what has been previously observed in other studies using Taz, where the degree of activation is greater than ten fold [27,28]. However, unlike our design, these studies measured the expression from the P_*ompC *_promoter. In the present work, we measured the expression from a downstream promoter, P_*luxI*_. Thus, there is an additional stage between the sensor and reporter in our design. Likely, expression of LuxR_Δ2-162 _from the P_*ompC *_promoter is not sufficiently strong to activate the P_*luxI *_promoter without further amplification. However, when we add amplification by including positive feedback, we then obtain robust expression.

## Conclusions

In this work, we developed a simple modular genetic amplifier based on a constitutively active variant of LuxR. We tested this amplifier by coupling it to a one-component tetracycline sensor and a two-component aspartate sensor. In both instances, the amplifier was able to increase the dynamic range and sensitivity of the integrated circuit. Based on these results, this amplifier most likely can be coupled to any cell-based sensor where the output involves the transcription of a gene. In these regards, we have successfully constructed a reusable component.

In addition to sensing applications, the amplifier can also be used to create devices of greater complexity in function. One intriguing application concerns impedance matching. Impedance mismatch occurs when the output range of one sub-circuit does not match the input range of another sub-circuit to which it is connected. To effectively link these two sub-circuits, the respective output and input ranges should match one another. As positive feedback can significantly alter the response of a sub-circuit, it can be used as an 'impedance matching' device by coupling two different sub-circuit circuits together that have disparate requirements for signal levels to operate correctly.

A primary goal of synthetic biology is to design modular components with defined behavior that can be reused in diverse applications [29-32]. The ideal component should have predicable behavior regardless of the context in which it is applied. This is a significant challenge. Even in our experiments, while we rightly hypothesized that we would see amplification due to the positive feedback, we see a different response when we coupled the amplifier to the two different sensors. For instance, the tetracycline sensor showed a major increase in sensitivity but only moderate increase in the dynamic response. The aspartate sensor, however, showed a major increase in the dynamic response but only a moderate increase in sensitivity. Moreover, the amplifier increased background expression in the case of the aspartate sensor but not in the case of the tetracycline sensor. The origins of these differences are unknown, but may arise due to variations, for example, in plasmid copy number, promoter strengths, and the metabolic burden imposed by each circuit. While further engineering can be used to control for these individual factors, their effects are often non-trivial to isolate and quantify.

## Competing interests

The authors declare that they have no competing interests.

## Authors' contributions

CR and KB conceived the experiments. GN and KW performed experiments. CR and KB wrote the manuscript. All authors read and approved the final manuscript.
